# MTBseq-nf: Enabling Scalable Tuberculosis Genomics “Big Data” Analysis Through a User-Friendly Nextflow Wrapper for MTBseq Pipeline

**DOI:** 10.3390/microorganisms13122685

**Published:** 2025-11-25

**Authors:** Abhinav Sharma, Davi Josué Marcon, Johannes Loubser, Karla Valéria Batista Lima, Gian van der Spuy, Emilyn Costa Conceição

**Affiliations:** 1 SAMRC Centre for Tuberculosis Research, Division of Molecular Biology and Human Genetics, Faculty of Medicine and Health Sciences, Stellenbosch University, Cape Town 7505, South Africa; abhinavsharma@sun.ac.za (A.S.); jloubser@sun.ac.za (J.L.); 2 Pós-Graduação em Biologia Parasitária na Amazônia, Instituto de Ciências Biológicas e da Saúde, Universidade do Estado do Pará, Belém 66095-015, Pará, Brazil; davijosuemarcon@gmail.com (D.J.M.); karlavaleria2007@gmail.com (K.V.B.L.); 3 Seção de Bacteriologia e Micologia, Instituto Evandro Chagas, Ananindeua 67030-000, Pará, Brazil

**Keywords:** bioinformatics pipeline, genomic surveillance, MTBseq, *Mycobacterium tuberculosis*, Nextflow, tuberculosis genomics, whole-genome sequencing, workflow

## Abstract

The MTBseq pipeline, published in 2018, was designed to address bioinformatics challenges in tuberculosis (TB) research using whole-genome sequencing (WGS) data. It was the first publicly available tool on GitHub to perform full analysis of WGS data for *Mycobacterium tuberculosis* complex (MTBC) encompassing quality control through mapping, variant calling for lineage classification, drug resistance prediction, and phylogenetic inference. However, the pipeline’s architecture is not optimal for analyses on high-performance computing or cloud computing environments that often involve large datasets. To overcome this limitation, we developed MTBseq-nf, a Nextflow wrapper that provides parallelization for faster execution speeds in addition to several other significant enhancements. The MTBseq-nf wrapper can run several instances of the same step in parallel, fully utilizing the available resources, unlike the linear, batched analysis of samples in the TBfull step of the MTBseq pipeline. For evaluation of scalability and reproducibility, we used 90 *M. tuberculosis* genomes (European Nucleotide Archive—ENA accession PRJEB7727) for the benchmarking analysis on a dedicated computational server. In our benchmarks, MTBseq-nf in its parallel mode is at least twice as fast as the standard MTBseq pipeline for cohorts exceeding 20 samples. Through integration with the best practices of nf-core, Bioconda, and Biocontainers projects MTBseq-nf ensures reproducibility and platform independence, providing a scalable and efficient solution for TB genomic surveillance.

## 1. Introduction

Next-generation sequencing (NGS) has revolutionized tuberculosis (TB) research, diagnosis, and surveillance; however, analyzing such voluminous amounts of complex bioinformatics data, presents numerous computational challenges [1]. The MTBseq pipeline was published in 2018 to address some of the challenges of data-analysis and improve the reproducibility of whole-genome sequencing (WGS) analysis of *Mycobacterium tuberculosis* complex (MTBC) [2].

MTBseq (hereafter standard MTBseq or MTBseq-standard) was one of the first publicly available comprehensive end-to-end pipelines and is widely used by researchers working with WGS data to analyze data and generate strain classification, phylogenetic trees, mapping and variant statistics, Single Nucleotide Polymorphism (SNP) distance matrix, and cluster groups [2]. On the other hand, the computational infrastructure available to researchers ranges from the traditional, in-house servers to high-performance computing (HPC) platforms.

The available options have diversified in recent years with the development of (i) batch computing functionality by cloud computing vendors such as Oracle, Amazon Web Services (AWS), Google, Microsoft Azure, International Business Machine (IBM), and Alibaba, etc., and (ii) open source job orchestrators like Kubernetes, Apache Meson, and Hashicorp Nomad to analyze the exponentially increasing volumes of data in an efficient and secure manner [3,4] for precision public health [5] and precision medicine [6].

This adoption trend of NGS technologies and use of modern job orchestrators is expected to continue for the foreseeable future, especially in the context of precision public health and precision medicine. The MTBseq pipeline, in its current form performs sub-optimally for these modern computing environments; therefore, we aimed to enhance the MTBseq pipeline for traditional as well as modern job orchestrators utilized in large-scale genomic analysis, while optimizing the costs and making the analysis time predictable.

We developed MTBseq-nf, a wrapper built with the Nextflow workflow engine [7] to provide an alternative to current users of MTBseq-standard with various enhancements in its user-friendliness, maintainability, reproducibility, and scalability for large-scale genomic data analysis [8].

## 2. Materials and Methods

### 2.1. The Design of MTBseq (Standard) Pipeline

**Figure 1:**
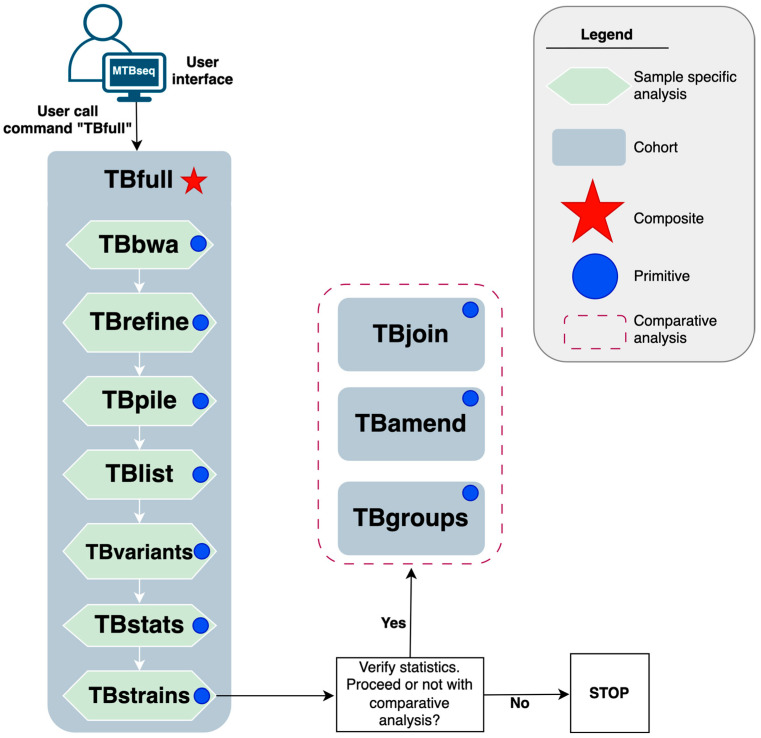
A schematic diagram of the MTBseq-standard pipeline by Kohl et al. (2018) [2], illustrating sequential dependency of analytical steps.

The MTBseq-standard pipeline by Kohl et al. [2] relies upon underlying tools such as GATK3 [9], PICARD [10], BWA [11], and SAMTOOLS [12]; and glues these tools together using the perl5 [13] programming language as shown in Figure 1.

Its execution model is built upon core abstractions (called steps) such as reference mapping (TBbwa) and variant calling (TBvariants). These steps can be broadly classified into two groups based on whether they process a single sample at a time (e.g., TBbwa, TBvariants) or the entire batch (e.g., TBamend, TBgroups). The former are amenable to parallelisation whereas the latter are not. In addition, MTBseq-standard also combines several primitive steps into a composite step (TBfull) which may be called as a single entity to facilitate routine usage as summarized in Figure 1.

### 2.2. Implementation of MTBseq-nf Wrapper

We developed MTBseq-nf wrapper, on top of the open-source MTBseq pipeline, and implemented numerous enhancements across four broad themes (i) user-friendliness, (ii) scalability, (iii) reproducibility, and (iv) maintainability, as summarized in Table 1.

In the implementation of the MTBseq-nf wrapper pipeline we built upon two core components. Firstly, the MTBseq-standard pipeline Kohl et al. [2] exposes a --step parameter, on the command line, to allow users to dictate which step of the pipeline they wish to initiate. Figure 1 highlights how the MTBseq-standard pipeline facilitates the usage of sequential sample-specific steps for routine executions, through a composite TBfull step that automates the execution of sample-specific primitive steps from the TBbwa step until the TBstrains step. Upon completion of these steps, the users are expected to provide a TSV file, containing a list of samples and corresponding library names, that should be included in further comparative analysis steps such as TBjoin, TBamed, and TBgroup generating the principal outputs of the pipeline as described in the Supplemental Data.

Secondly, the use of Nextflow workflow manager as per nf-core best-practices pipeline template. Nextflow is a workflow management system designed to address the challenges of high-throughput NGS data at scale in a reproducible manner [7], allowing researchers to create complex workflows that integrate multiple bioinformatics tools into a single cohesive workflow while maintaining portability across different computing infrastructures.

The nf-core community further enhances the Nextflow ecosystem by bringing together Nextflow users through a Slack group, hackathons, seminars, training, and other community initiatives that foster collaboration and knowledge sharing [14,15]. Moreover, the nf-core pipeline template is constructed with rigorous standards to guarantee robustness, portability, and user-friendliness, due to its integration with other projects from the nf-core ecosystem, such as nf-core/configs and nf-core/modules.

**Figure 2:**
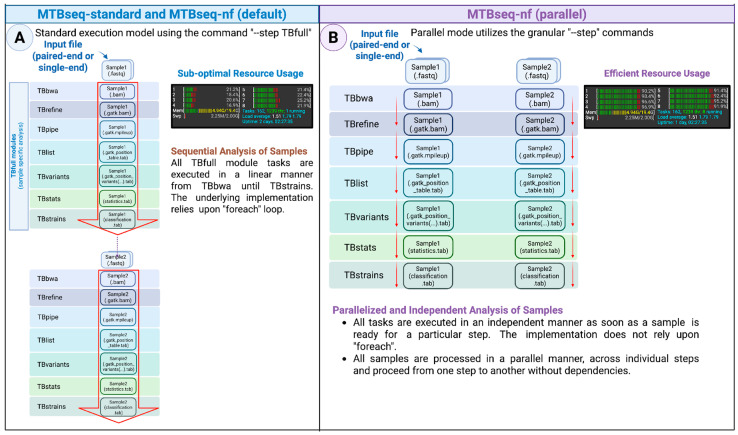
An overview of the (**A**) MTBseq-standard, MTBseq-nf (default) with linear and batched analysis and (**B**) the MTBseq-nf (parallel) mode, that allows an individual sample to continue to the next steps, independent of other samples.

Internally, the MTBseq-standard pipeline depends on the sequential analysis of input sequences through the foreach looping structures, with each sample advancing to the subsequent step only after all FASTQ samples have completed an individual step (Figure 2A). On the other hand, the MTBseq-nf wrapper pipeline has two execution modes: (i) default and (ii) parallel mode, activated by the --parallel parameter on the command line. Both models are functionally equivalent to the combination of TBfull, TBgroups, TBamend, and TBjoin steps of the MTBseq pipeline.

The principal insights we employed to implement the parallel mode was to combine MTBseq’s modular stepwise architecture exposed through the --step parameter with the inherent task parallelization provided by the Nextflow workflow engine.

Consequently, when executed with the --parallel parameter, the MTBseq-nf wrapper pipeline employs the exact same primitive steps utilized by the TBfull step. This optimizes the movement of intermediate files through the individual steps (Figure 2B), resulting in a significant reduction (>50%) in the overall execution time of the pipeline, especially when the number of samples in a cohort are larger (>20 samples).

We depend on the fact that when a sample-specific step is executed with a single sample, the foreach loop iterates only once, since only a single sample is accessible to that particular step. Moreover, the MTBseq-nf pipeline (in both modes) automates the generation of a TSV file when all samples undergo the initial phases of the TBfull step and initiates the comparative analysis stage. This mirrors the behavior of the MTBseq-standard pipeline for subsequent steps, minimizing the manual intervention by users.

### 2.3. Validation Infrastructure and Dataset

Our computational experiments were conducted using a virtual server on the Oracle Cloud Infrastructure that had 32 CPUs (equivalent to 16 Oracle CPUs), 64 GB of RAM, and a 2 TB boot disk. The essential software prerequisites included (i) the Java programming language [16], (ii) Nextflow [7], and (iii) Docker [17]. The MTBseq-standard pipeline v1.1.0 was set up utilizing the bioconda recipe file provided in the Supplemental Data.

The choice of using a server was crucial to our investigation of execution runtime, given that HPC queue systems introduce an unpredictable delay prior to the execution of a submitted compute job. The Docker platform was selected for its widespread application in cloud batch computing environments (AWS Batch, Azure Batch, and Google Batch) and modern container orchestration systems such as Kubernetes and Hashicorp Nomad.

The choice of Docker containers was also informed by the resources from the upstream biocontainer project [18], which originate from bioconda recipes [19]. This method allowed us to assess the risk of non-reproducibility due to infrastructural inconsistencies and facilitated an isolated study of the major outcomes of the pipeline.

We relied upon the dataset used in the original study [2] to evaluate the reproducibility and scalability of the MTBseq-nf wrapper in terms of growth of execution time versus the cohort size as well as the validity of results produced. The dataset, as described by Schleusener et al. (2017) [20] is publicly available under the European Nucleotide Archive (ENA) accession code PRJEB7727. Furthermore, Kohl et al. [2] analyzed 91 samples for resistance and lineage profiling in a comparative setting as part of Supplementary Materials of the original publication. These identifications (IDs) are shared in the Supplemental Data accompanying this manuscript.

The data provided to ENA comprises 133 paired-end FASTQ files derived from cultured *M. tuberculosis* samples (Supplemental Data), and upon meticulous examination, we observed certain anomalies. Firstly, some samples had several matching experiment-accession entries. The Supplemental Data provides a frequency count of secondary-sample-accession pertaining to experiment-accession ERS IDs. Secondly, the samples used by Kohl et al. (2018) [2] exhibited discrepancies relative to those documented in the ENA project PRJEB7727. Thirdly, one sample, ERS457325, had no record of any associated files in the specified ENA project. Therefore, to mitigate the influence of confounding variables, we chose to deduplicate the samples rather than merge those with similar accession numbers.

This was performed by using the initial occurrence of secondary-sample-accession IDs from the files obtained from the ENA project and by omitting the absent ERS457325 (4730-03) from our analysis. This reduced the final dataset to 90 paired-end FASTQ files. The final sample sheet is included in the Supplemental Data.

**Figure 3:**
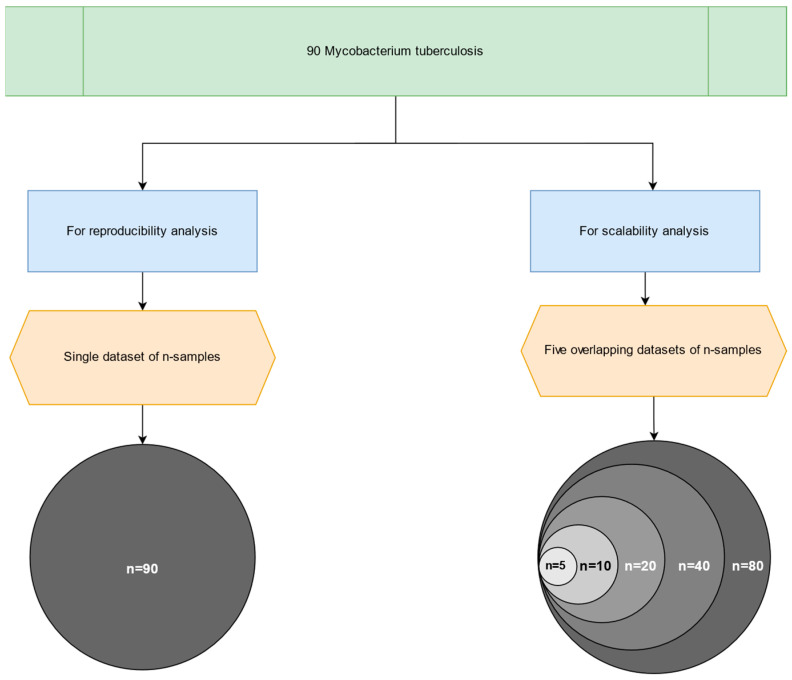
An overview of the six subsets, used for the scalability analysis—from the original publication by Kohl et al. (2018) [2].

For the scalability study, we partitioned the dataset into six cohorts with an incremental number of samples, namely 5, 10, 20, 40, 80, and lastly 90 FASTQ paired-end files, as illustrated in Figure 3. Additionally, the largest dataset was used to validate the primary outputs produced by the MTBseq and MTBseq-nf (default and parallel) pipelines.

### 2.4. Experimental Set Up for Evaluation of Scalability and Reproducibility

We conducted experiments comparing MTBseq-nf in both default and parallel modes with the MTBseq-standard pipeline in triplicates. This comprised (i) an intra-modal analysis to assess reproducibility within each mode, and (ii) an inter-modal analysis to assess reproducibility across distinct modes. This resulted in nine distinct runs of the pipelines which are highlighted in the Supplemental Data.

The parameters utilized for these experiments and the associated results are published as part of the Zenodo repository https://doi.org/10.5281/zenodo.14678756 (17 January 2025), while the names of individual runs and significant parameters are reported in the Supplemental Data. Moreover, each experiment was conducted independently without employing Nextflow’s resume feature to guarantee fresh execution of each individual step.

To evaluate the scalability of the pipeline, we analyzed the execution time increase for the six specified dataset sizes using the default mode and parallel mode of MTBseq-nf on these datasets. The executions of MTBSeq-nf in both default and parallel modes were monitored and visualized via the Seqera Platform [21], which serves as a centralized repository for tracking execution metrics and maintaining pipeline configurations and helps democratize the bioinformatics expertise and tools for non-experts [22].

To evaluate the reproducibility of the different modes of the MTBseq-nf, we compared the principal results of inter- and intra-modal triplicated experiments by conducting three-way diffs using Araxis Merge [23] and range analysis of numerical data using R language [24], along with a phylogenetic tree using the IQTREE program (v2.3.4) [25] as summarized in the Supplemental Data.

We used a three-way visual diff to assess the disparities in the principal results, which are qualitative in nature. For the mapping and variant statistics produced by TBstats (including Mapped Reads and SNPs), we used range statistics (min-max analysis) in R (v4.3.1) to estimate the relative changes across various intra- and inter-modal studies. The scripts employed for the analysis and visualizations are available in the following repository: https://github.com/abhi18av-phd-projects/mtbseq-nf-publication-analysis (accessed on 18 November 2025).

## 3. Results

### 3.1. Thematic Improvements in MTBseq-nf

MTBseq-nf offers substantial enhancements over MTBseq-standard, including an innovative parallel mode that significantly reduces overall execution time for large cohorts. The implemented features span four key themes: (1) user-friendliness, (2) scalability, (3) reproducibility, and (4) maintainability, as highlighted in Table 1 and detailed in the Supplemental Data (Table S2).

MTBseq-nf leverages the standardized template from the nf-core community, providing numerous advantages including: (i) configuration files (dotfiles) that address code quality, linting, and testing requirements; (ii) integration with the nf-core/configs project for portability across institutional infrastructures; (iii) access to well-tested modules from the nf-core/modules project; and (iv) a graphical user interface through the nf-schema project for users less familiar with command-line operations (Supplemental Data S1).

### 3.2. Reproducibility Analysis of Intra-Modal Comparison

For the intra-modal comparison of the three pipeline-mode combinations, we conducted triplicated experiments with identical infrastructure, dataset, and parameters as described in Supplemental Data S8.

As summarized in Table 2, the classification and SNP distance matrix results demonstrated consistency across experiments, with differences owing to the date of execution of the experiment, which was expected, as successive runs could only begin after previous runs were completed. Similarly, the phylogenetic trees remained stable across different experiments. The transmission cluster groups, which are assigned labels based on the SNP matrix, showed some variation in exact labeling across different runs of the TBgroups step, attributable to the inherent nature of the agglomerative algorithm used, though the actual grouping of samples remained accurate.

**Figure 4:**
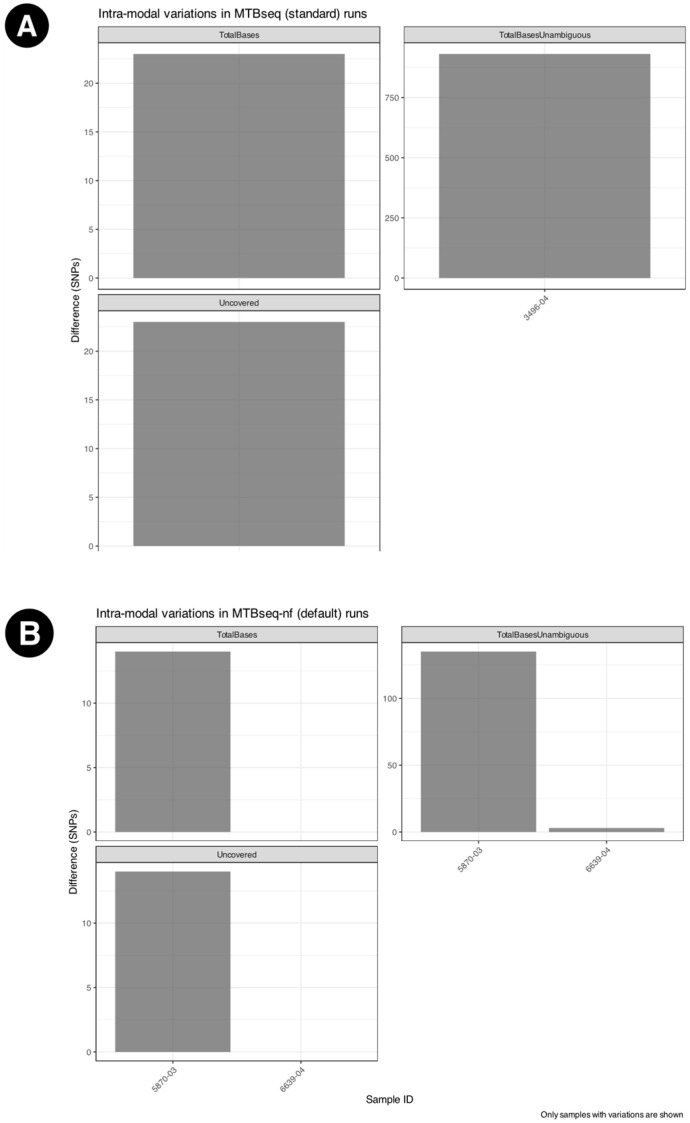
Intra-modal variations across triplicated runs for (**A**) MTBseq-standard, observed only for three samples (**B**) MTBseq-nf (default) across triplicated runs, observed only for two samples. No differences were observed across different runs of the MTBseq-nf parallel mode.

Among the intra-modal comparisons, Figure 4 some numeric fields in the MTBseq statistics report showed differences in triplicated runs of the MTBseq-nf default mode and MTBseq-standard, as shown in Figure 4A and Figure 4B, respectively. However, for MTBseq-nf parallel mode, we did not observe variations across any of the numerical fields indicating complete reproducibility of results in the parallel mode of the MTBseq-nf pipeline.

### 3.3. Reproducibility Analysis of Inter-Modal Comparison

**Figure 5:**
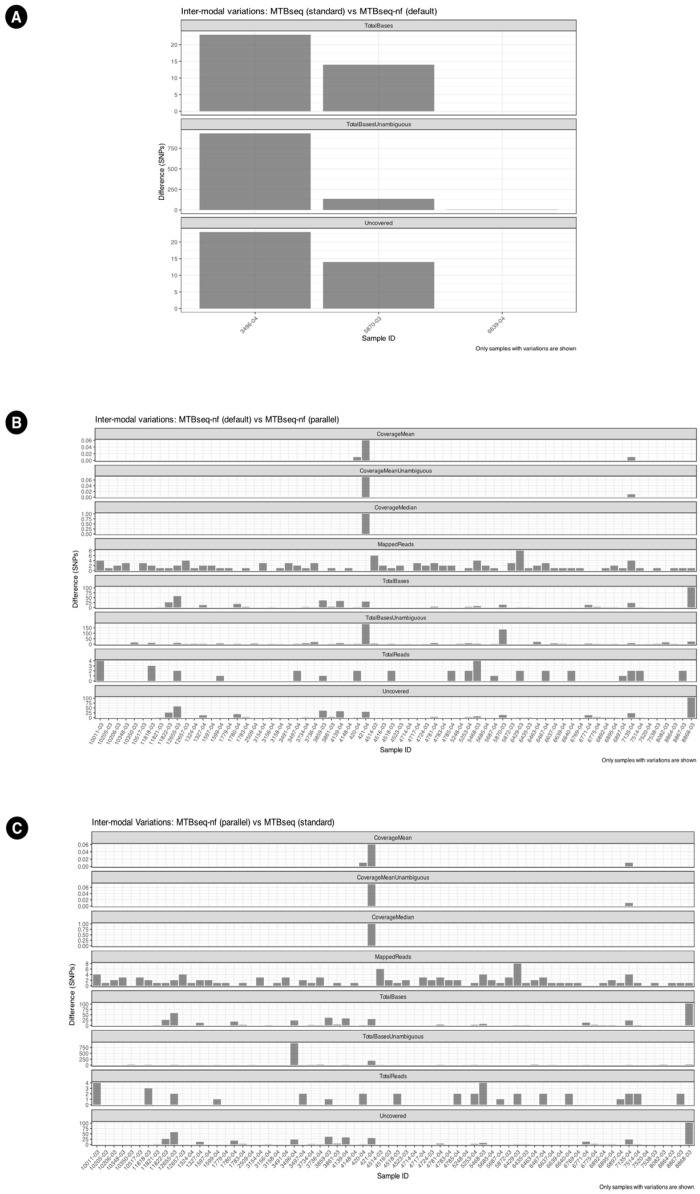
Inter-modal variations between (**A**) MTBseq-standard and MTBseq-nf (default), wherein only 3 samples have variations. (**B**) MTBseq-nf (default) and MTBseq-nf (parallel). (**C**) MTBseq-nf (parallel) and MTBseq-standard.

**Figure 6:**
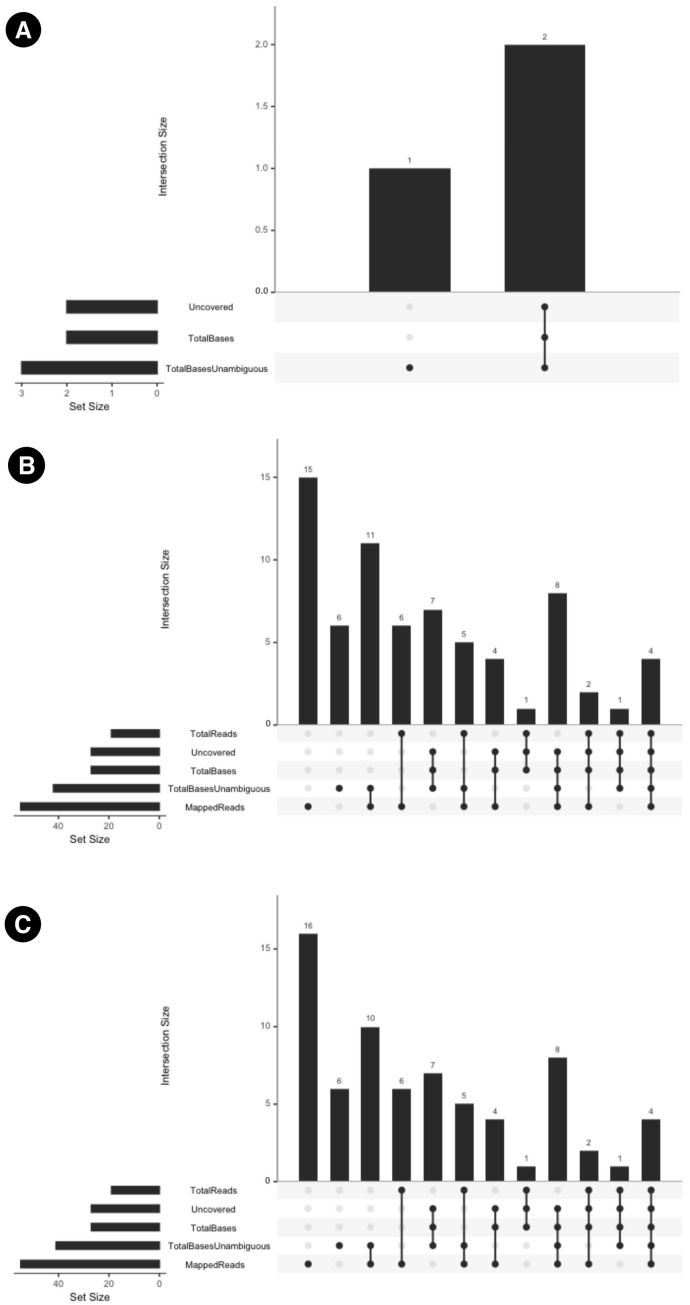
UpSet plots which summarize the inter-modal variations between (**A**) MTBseq-standard and MTBseq-nf (default). (**B**) MTBseq-nf (default) and MTBseq-nf (parallel). (**C**) MTBseq-nf (parallel) and MTBseq-standard.

The inter-modal variation has been summarized in Figure 5. Additionally, the UpSet plots in Figure 6 illustrate the variation in specific columns and the corresponding number of samples. MTBseq-standard vs. MTBseq-nf (default). Figure 5A highlights the numerical variation in SNP counts between MTBseq-standard and MTBseq-nf (default) runs across the fields of Uncovered, TotalBasesUnambguous, and TotalBases and demonstrates that the range of variation between MTBseq-standard and MTBseq-nf (default) is very small.

When comparing MTBseq-nf (default) vs. MTBseq-nf (parallel) in Figure 5B, we observed variations similar to the comparison of MTBseq-nf (parallel) vs. MTBseq-standard in Figure 5C, despite the latter being functionally equivalent to MTBseq-nf (default). This indicates that the MTBseq-nf parallel mode is on par with the MTBseq-standard and MTBseq-nf as the differences are fractional compared to the overall range of value per numeric column.

### 3.4. Scalability Analysis of MTBseq-nf (Default) and MTBseq-nf (Parallel)

**Figure 7:**
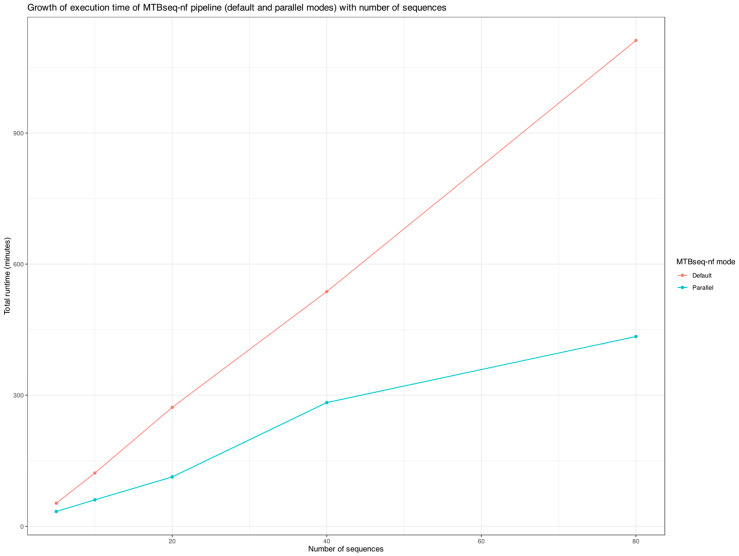
The increase in total runtime of the MTBseq-nf parallel mode and default mode versus the sample size.

To evaluate execution time, we included only one specific run for each mode of MTBseq-nf and excluded MTBseq-standard from the analysis due to the manual intervention step required after the TBfull step and prior to the TBstats, TBgroups, and TBamend steps. Since MTBseq-standard and MTBseq-nf (default) are functionally equivalent, comparing total execution time between MTBseq-nf (default) and MTBseq-nf (parallel) was sufficient to analyze the growth curves in Figure 7.

As highlighted in Figure 7, when processing more than 10 FASTQ paired-end files, the execution time of MTBseq-nf (default) is at least double that of MTBseq-nf (parallel), highlighting the significant performance benefits of the parallel mode optimizations. This difference becomes increasingly pronounced as the sample size increases (>20 samples), making MTBseq-nf (parallel) particularly valuable for large-scale genomic analyses requiring substantially less time (<50%).

## 4. Discussion

The MTBseq-nf pipeline represents a significant effort to optimize and modernize the widely adopted MTBseq-standard pipeline for analyzing WGS data from the *M. tuberculosis* culture. In the process, we also implement various enhancements to improve the user experience, especially in high-throughput and diverse computing environments.

One of the major advancements introduced by MTBseq-nf is the optional parallel execution mode. By decoupling sequential dependencies and capitalizing on the Nextflows inherent parallelization, MTBseq-nf significantly reduces the overall runtime of the pipeline. Our empirical analysis indicated that the execution time scales gracefully in the parallel mode, specifically as the number of samples increases—a critical feature for genomic surveillance programs processing hundreds or thousands of isolates regularly.

The task-level isolation introduced by parallel mode ensures that each primitive step has sufficient memory (and CPU) during its execution, and as soon as the step completes, the relevant docker container exits and the docker container engine ensures that the computational resources of the host system are released and made available for other queued tasks [26]. In the TBfull steps within MTBseq and MTBseq-nf (default), the consumed memory is not immediately released when the previous process completes as the computations are performed sequentially within the same container. The subsequent processes, therefore, have slightly reduced access to memory, which may impact the memory-intensive computations required by GATK.

This improvement with MTBseq-nf parallel is especially beneficial for the integration of bioinformatic analysis in routine diagnostics and surveillance work, chiefly in resource-constrained environments such as low- and middle-income countries (LMICs), where TB is endemic and timely analysis is vital, and where infrastructure may not be suited to long, sequential compute jobs. Furthermore, the ability to execute the MTBseq-nf pipeline across laptops, servers, HPC clusters, and cloud batch systems allows for unprecedented flexibility and broader adoption.

Reproducibility is a cornerstone of bioinformatics tools, chiefly when used in clinical and public health contexts. MTBseq-nf leverages Nextflow’s declarative parameters feature, integration with container systems [27] such as Docker, and in-built caching mechanisms to rapidly deliver reproducible results across varied environments. The triplicated intra-modal experiments confirmed the consistency of principal outputs across different runs of MTBseq-nf, particularly in the parallel mode, which showed no measurable variation in statistics or results.

Users benefit from the baseline improvements within the MTBseq-nf (default) mode, without making use of the parallel feature. Results obtained from intra-modal (Figure 4A,B) analysis as well inter-modal analysis (Figure 5A and Figure 6A) of MTBseq-nf (default) and MTBseq-standard, confirmed their functional equivalence.

Moreover, discrepancies in output observed between sequential and parallel executions, such as minor differences in TBstats reports, are likely due to the memory-intensive behavior of tools like GATK when executed sequentially on all samples in shared containers. In contrast, the task-level isolation in the parallel mode ensures more consistent memory allocation, leading to increased stability of results across executions.

In architecting MTBseq-nf on top of MTBseq-standard, we have prioritized a straight-forward user experience. By adopting the nf-core best practices, users benefit from structured configuration files, standardized output, and simplified parameter management. Features like an explicit sample sheet, an optional Graphic User Interface (GUI) through the nf-schema project, and remote execution monitoring and infrastructure management (via the Seqera platform) [21] further enhance usability, especially for users less familiar with the command line.

Another valuable addition is the inclusion of modules like FastQC and MultiQC [28], which offer quality control summaries that are now seamlessly integrated into the pipeline without manual intervention. In contrast, integrating such tools into MTBseq-standard would require creation, customization, and testing of Perl-wrapped modules.

The adoption of the nf-core pipeline template ensures the future viability of MTBseq-nf by allowing rapid integration of updates and modules. The traditional software architecture of the MTBseq-standard pipeline makes maintenance and extension daunting, notably for newcomers to the project.

In contrast, the modular design of MTBseq-nf, owing to the nf-core best-practices template, aligns with modern software engineering practices, including continuous integration and delivery (CI/CD), unit testing (e.g., using nf-test), and community contributions via GitHub (online service). This design ensures that MTBseq-nf can evolve in response to user needs, bug reports, and new tool integrations, such as IQTREE, which, although not currently included, can be added as an integrated module in future iterations depending on research requirements and user-engagement.

Additionally, in terms of results, minor variations in SNP clustering labels across runs are an artifact of the agglomerative clustering algorithm. These variations do not impact downstream phylogenetic analyses but underscore the importance of clear documentation and careful interpretation of clustering outputs. Furthermore, the versatility of MTBseq-nf enables its deployment in both high-resource and constrained settings, supporting TB control efforts globally. By simplifying complex analyses and promoting reproducibility, the pipeline aligns with the broader goals of precision public health and genomic epidemiology.

In terms of limitations, MTBseq-nf (i) inherits the core computational logic of MTBseq-standard, limiting opportunities for deeper architectural optimization and (ii) the reliance on MTBseq-standards’ Perl-based modules pose a hard-limit on how extensively the workflow can be modernized without altering the Perl code.

Future work may involve (i) translating these steps into native Nextflow processes or subworkflows, improving maintainability, and supporting pluggable alternative tools for the individual steps such as alignment and variant calling tools. Another interesting area to explore is (ii) computational carbon footprints, an increasingly relevant consideration in bioinformatics research [29,30] by comparing carbon emissions as well as (iii) total cost of analysis for large scale bioinformatics analysis for the construction of epidemiological databases and portals.

## 5. Conclusions

MTBseq-nf modernizes the MTBseq pipeline by enabling scalable, reproducible, and efficient analysis of *M. tuberculosis* WGS data. Its integration with Nextflow and nf-core standards enhances performance and portability across computing environments, supporting both high-resource and low-resource settings in LMIC settings for genomic surveillance, in a cost-effective manner.

## Figures and Tables

**Table 1:** Summary of enhancements (features) in MTBseq-nf, as compared to the original MTBseq-standard pipeline, spanning four distinct categories.

Theme	Feature
User-friendliness	Ease of download
User-friendliness	Explicit samplesheet
User-friendliness	Graphical user interface
User-friendliness	MultiQC Summary report
User-friendliness	CSV and TSV format cleanup
User-friendliness	Remote monitoring
User-friendliness	Manual steps
User-friendliness	Flexible output location
Maintainability	Extensibility
Maintainability	Module testing
Maintainability	Test dataset
Scalability	Parallel execution
Scalability	HPC compatibility
Scalability	Resource allocation
Scalability	Dynamic retries
Scalability	Execution cache
Scalability	Reduced data footprint
Scalability	Reduced cloud computing costs
Reproducibility	Declarative parameters file
Reproducibility	Portability
Reproducibility	Save intermediate files

**Table 2:** Summary of intra-modal analysis of principal outputs of triplicated runs.

Principal Output	MTBseq (Standard)	MTBseq-nf (Default Mode)	MTBseq-nf (Parallel Mode)
Classification	No differences	No differences	No differences
SNP distance matrix	No differences	No differences	No differences
Phylogenetic tree	No differences	No differences	No differences
Cluster groups	Consistent agglomeration	Consistent agglomeration	Consistent agglomeration
Statistics	Minor differences	Minor differences	No differences
Classification	No differences	No differences	No differences

## Data Availability

The Zenodo record https://doi.org/10.5281/zenodo.14678756 contains results and metadata for the nine runs of MTBseq-standard, MTBseq-nf (default), and MTBseq-nf (parallel) including (i) MultiQC reports, (ii) runtime metrics, (iii) pipeline parameters, and (iv) results of all experiments. The codes are available in the following links: MTBseq-nf pipeline https://zenodo.org/records/15234640; MTBseq-nf analysis scripts https://github.com/abhi18av-phd-projects/pub-mtbseq-nf. The dataset, as described by Schleusener et al. (2017) [20]—is publicly available under the European Nucleotide Archive (ENA) accession code PRJEB7727.

## Supplementary Materials

The following supporting information can be downloaded at: https://www.mdpi.com/article/10.3390/microorganisms13122685/s1, S-1 Auto-generated graphical user interface for the MTBseq-nf pipeline on Seqera Platform (formerly Nextflow Tower). S-2 Summary of validation technique used for principal results. S-3 Validation dataset: (1) S-3.01 Samplesheet ERS (from original publication Kohl et al., 2018 [2]—Supplementary Table S2); (2) S-3.02 Report from ENA https://www.ebi.ac.uk/ena/browser/view/PRJEB7727. (3) S-3.03 Frequency of ERS IDs from original publication; (4) S-3.04 Final samplesheet used for the analysis; (5) S-3.05 Metadata report for the Bioproject, generated using nf-core/fetchngs. S-4 Bioconda recipe for MTBseq v1.1.0: MTBseq_conda_recipe.yml. S-5 An overview of key enhancements in MTBseq-nf, Nextflow wrapper for the original MTBseq pipeline. S-6 Intra-modal analysis, with 3-way HTML diff reports generated by Araxis merge software. (1) S-6.01 intra-modal-araxiscompare-pub-90samples-mtbseq-nf-parallel-runs-classification.html; (2) S-6.02 intra-modal-araxiscompare-pub-90samples-mtbseq-nf-parallel-runs-cluster-groups.html; (3) S-6.03 intra-modal-araxiscompare-pub-90samples-mtbseq-nf-parallel-runs-snp-matrix.html; (4) S-6.04 intra-modal-araxiscompare-pub-90samples-mtbseq-nf-parallel-runs-statistics.html; (5) S-6.05 intra-modal-araxiscompare-pub-90samples-mtbseq-nf-runs-classification.html; (6) S-6.06 intra-modal-araxiscompare-pub-90samples-mtbseq-nf-runs-cluster-groups.html; (7) S-6.07 intra-modal-araxiscompare-pub-90samples-mtbseq-nf-runs-snp-matrix.html; (8) S-6.08 intra-modal-araxiscompare-pub-90samples-mtbseq-nf-runs-statistics.html; (9) S-6.09 intra-modal-araxiscompare-pub-90samples-mtbseq-standard-runs-classification.html; (10) S-6.10 intra-modal-araxiscompare-pub-90samples-mtbseq-standard-runs-cluster-groups.html; (11) S-6.11 intra-modal-araxiscompare-pub-90samples-mtbseq-standard-runs-snp-matrix.html; (12) S-6.12 intra-modal-araxiscompare-pub-90samples-mtbseq-standard-runs-statistics.html. S-7 Growth of total execution time of different modes of MTBseq-nf for 5 datasets with increasing cohort size. S-8 Summary of different executions of MTBseq and MTBseq-nf in the triplicated set of experiments.

## Abbreviations

The following abbreviations are used in this manuscript:

AWS	Amazon Web Services
BWA	Burrows-Wheeler Aligner
CNPq	Conselho Nacional de Desenvolvimento Científico e Tecnológico (Brazilian Nationa Council for Scientific and Technological Development)
CPU	Central Processing Unit
CRediT	Contributor Role Taxonomy
DNA	Deoxyribonucleic Acid
Docker	Containerization platform
ENA	European Nucleotide Archive
ERS	ENA Experiment Accession Prefix
FASTQ	File format for sequencing reads; originally stands for “FASTA+Quality”
GATK	Genome Analysis Toolkit
GB	Gigabyte
GUI	Graphic User Interface
HPC	High-Performance Computing
IBM	International Business Machines
IDs	Identifications
IQ-TREE	Program for phylogenetic analysis
LMICs	Low- and Middle-Income Countries
MTBC	*Mycobacterium tuberculosis* Complex
MTBseq	*Mycobacterium tuberculosis* Sequencing Pipeline
MTBseq-nf	MTBseq Nextflow Wrapper Pipeline
MultiQC	Multi-Tool Quality Control Summary Software
*M. tuberculosis*	*Mycobacterium tuberculosis*
N/NE	Norte/Nordeste (North/Northeast)
NGS	Next-Generation Sequencing
nf-core	Community-curated Nextflow pipelines
NRF	National Research Foundation (South Africa)
Perl5	Programming Language version 5
PICARD	Picard Toolkit
PRJEB7727	ENA Project Accession Number
RAM	Random Access Memory
R	R Statistical Language
SAMTOOLS	Sequence Alignment/Map Tools
SNP	Single Nucleotide Polymorphism
TB	Tuberculosis
TBamend	MTBseq pipeline step
TBbwa	MTBseq pipeline step
TBfull	MTBseq pipeline step
TBgroups	MTBseq pipeline step
TBjoin	MTBseq pipeline step
TBstrains	MTBseq pipeline step
TBstats	MTBseq pipeline step
TBvariants	MTBseq pipeline step
TSV	Tab-Separated Values
WGS	Whole-Genome Sequencing
