# Supplementary material for: MTBseq-nf: Enabling Scalable Tuberculosis Genomics “Big Data” Analysis Through a User-Friendly Nextflow Wrapper for MTBseq Pipeline
Source: Microorganisms. 2025 Nov 25;13(12):2685. doi: 10.3390/microorganisms13122685 (PMC12734750; doi:10.3390/microorganisms13122685)
Supplement: Supplementary file 1 [file microorganisms-13-02685-s001.zip › microorganisms-3922628-supplementary/S-2-summary-validation-techniques.pdf]

Summary of validation techniques used for the different results generated by the MTBseq pipeline.

Report name	Validation technique
Classification	3-way diff report
SNP distance matrix	3-way diff report
Cluster groups	3-way diff report
Phylogenetic tree	Tree generated by IQTREE
Mapping and variant statistics	(i) 3-way diff report and (ii) statistical analysis
