# Supplementary material for: MTBseq-nf: Enabling Scalable Tuberculosis Genomics “Big Data” Analysis Through a User-Friendly Nextflow Wrapper for MTBseq Pipeline
Source: Microorganisms. 2025 Nov 25;13(12):2685. doi: 10.3390/microorganisms13122685 (PMC12734750; doi:10.3390/microorganisms13122685)
Supplement: Supplementary file 1 [file microorganisms-13-02685-s001.zip › microorganisms-3922628-supplementary/S-7-Growth-of-execution-time.pdf]

Growth of total execution time (runtime) of different modes of MTB-seq-nf for 5 datasets with increasing cohort size.

Number of samples	MTBseq-nf (default)	MTBseq-nf (parallel)
5	0h53m0s	0h34m0s
10	2h2m0s	1h0m45s
20	4h32m0s	1h53m0s
40	8h57m0s	4h43m0s
80	18h32m0s	7h14m0s
