# Supplementary material for: MTBseq-nf: Enabling Scalable Tuberculosis Genomics “Big Data” Analysis Through a User-Friendly Nextflow Wrapper for MTBseq Pipeline
Source: Microorganisms. 2025 Nov 25;13(12):2685. doi: 10.3390/microorganisms13122685 (PMC12734750; doi:10.3390/microorganisms13122685)
Supplement: Supplementary file 1 [file microorganisms-13-02685-s001.zip › microorganisms-3922628-supplementary/S-8-Nine-experiments.pdf]

Summary of different executions of MTBseq and MTBseq-nf in the triplicated set of experiments.

Run ID	Run name	Description	Notable non-default parameter
mtbseq-standard-run1	pub-90samples-mtbseq-standard-run1	MTBseq pipeline	
mtbseq-standard-run2	pub-90samples-mtbseq-standard-run2	MTBseq pipeline	
mtbseq-standard-run3	pub-90samples-mtbseq-standard-run3	MTBseq pipeline	
mtbseq-nf-run1	pub-90samples-mtbseq-nf-run1	MTBseq-nf pipeline	
mtbseq-nf-run2	pub-90samples-mtbseq-nf-run2	MTBseq-nf pipeline	
mtbseq-nf-run3	pub-90samples-mtbseq-nf-run3	MTBseq-nf pipeline	
mtbseq-nf-parallel-run1	pub-90samples-mtbseq-nf-parallel-run1	MTBseq-nf pipeline	–parallel
mtbseq-nf-parallel-run2	pub-90samples-mtbseq-nf-parallel-run2	MTBseq-nf pipeline	–parallel
mtbseq-nf-parallel-run3	pub-90samples-mtbseq-nf-parallel-run3	MTBseq-nf pipeline	–parallel
