# Supplementary material for: MTBseq-nf: Enabling Scalable Tuberculosis Genomics “Big Data” Analysis Through a User-Friendly Nextflow Wrapper for MTBseq Pipeline
Source: Microorganisms. 2025 Nov 25;13(12):2685. doi: 10.3390/microorganisms13122685 (PMC12734750; doi:10.3390/microorganisms13122685)
Supplement: Supplementary file 1 [file S-1-MTBSEQ_TOWER_SCHEMA.pdf › microorganisms-3922628-supplementary/S-1-MTBSEQ_TOWER_SCHEMA.pdf]

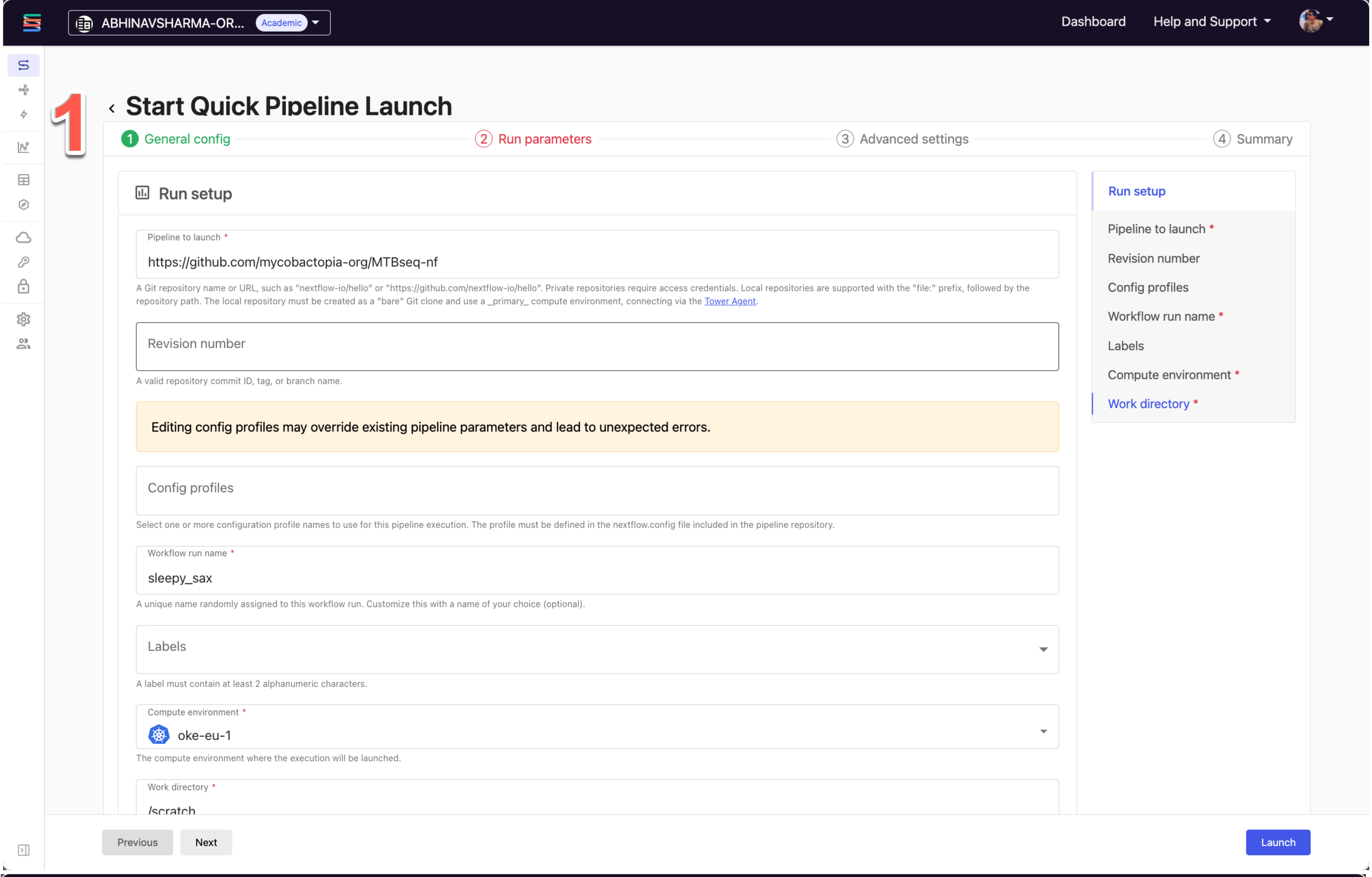
ABHINAVSHARMA-OR...
Academic
Dashboard
Help and Support
1
Start Quick Pipeline Launch
1 General config
2 Run parameters
3 Advanced settings
4 Summary
Run setup
Pipeline to launch *
https://github.com/mycobactopia-org/MTBseq-nf
A Git repository name or URL, such as "nextflow-io/hello" or "https://github.com/nextflow-io/hello". Private repositories require access credentials. Local repositories are supported with the "file:" prefix, followed by the repository path. The local repository must be created as a "bare" Git clone and use a _primary_ compute environment, connecting via the Tower Agent.
Revision number
A valid repository commit ID, tag, or branch name.
Editing config profiles may override existing pipeline parameters and lead to unexpected errors.
Config profiles
Select one or more configuration profile names to use for this pipeline execution. The profile must be defined in the nextflow.config file included in the pipeline repository.
Workflow run name *
sleepy_sax
A unique name randomly assigned to this workflow run. Customize this with a name of your choice (optional).
Labels
A label must contain at least 2 alphanumeric characters.
Compute environment *
oke-eu-1
The compute environment where the execution will be launched.
Work directory *
/scratch
Run setup
Pipeline to launch *
Revision number
Config profiles
Workflow run name *
Labels
Compute environment *
Work directory *
Previous
Next
Launch

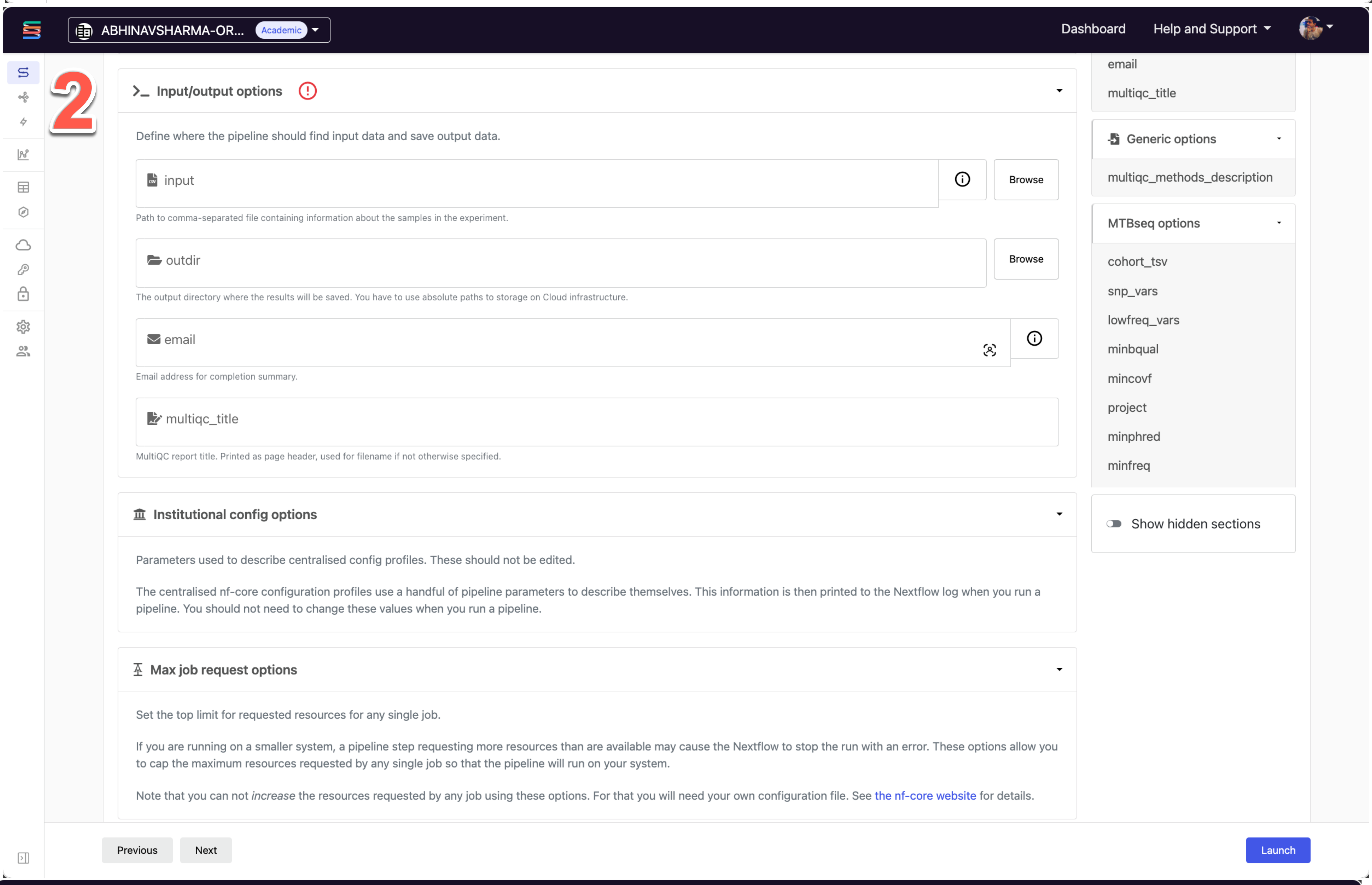
ABHINAVSHARMA-OR...
Academic
Dashboard
Help and Support
2
Input/output options
Define where the pipeline should find input data and save output data.
input
Browse
Path to comma-separated file containing information about the samples in the experiment.
outdir
Browse
The output directory where the results will be saved. You have to use absolute paths to storage on Cloud infrastructure.
email
Email address for completion summary.
multiqc_title
MultiQC report title. Printed as page header, used for filename if not otherwise specified.
Institutional config options
Parameters used to describe centralised config profiles. These should not be edited.
The centralised nf-core configuration profiles use a handful of pipeline parameters to describe themselves. This information is then printed to the Nextflow log when you run a pipeline. You should not need to change these values when you run a pipeline.
Max job request options
Set the top limit for requested resources for any single job.
If you are running on a smaller system, a pipeline step requesting more resources than are available may cause the Nextflow to stop the run with an error. These options allow you to cap the maximum resources requested by any single job so that the pipeline will run on your system.
Note that you can not increase the resources requested by any job using these options. For that you will need your own configuration files. See the nf-core website for details.
email
multiqc_title
Generic options
multiqc_methods_description
MTBseq options
cohort_tsv
snp_vars
lowfreq_vars
minbqual
mincovf
project
minphred
minfreq
Show hidden sections
Previous
Next
Launch

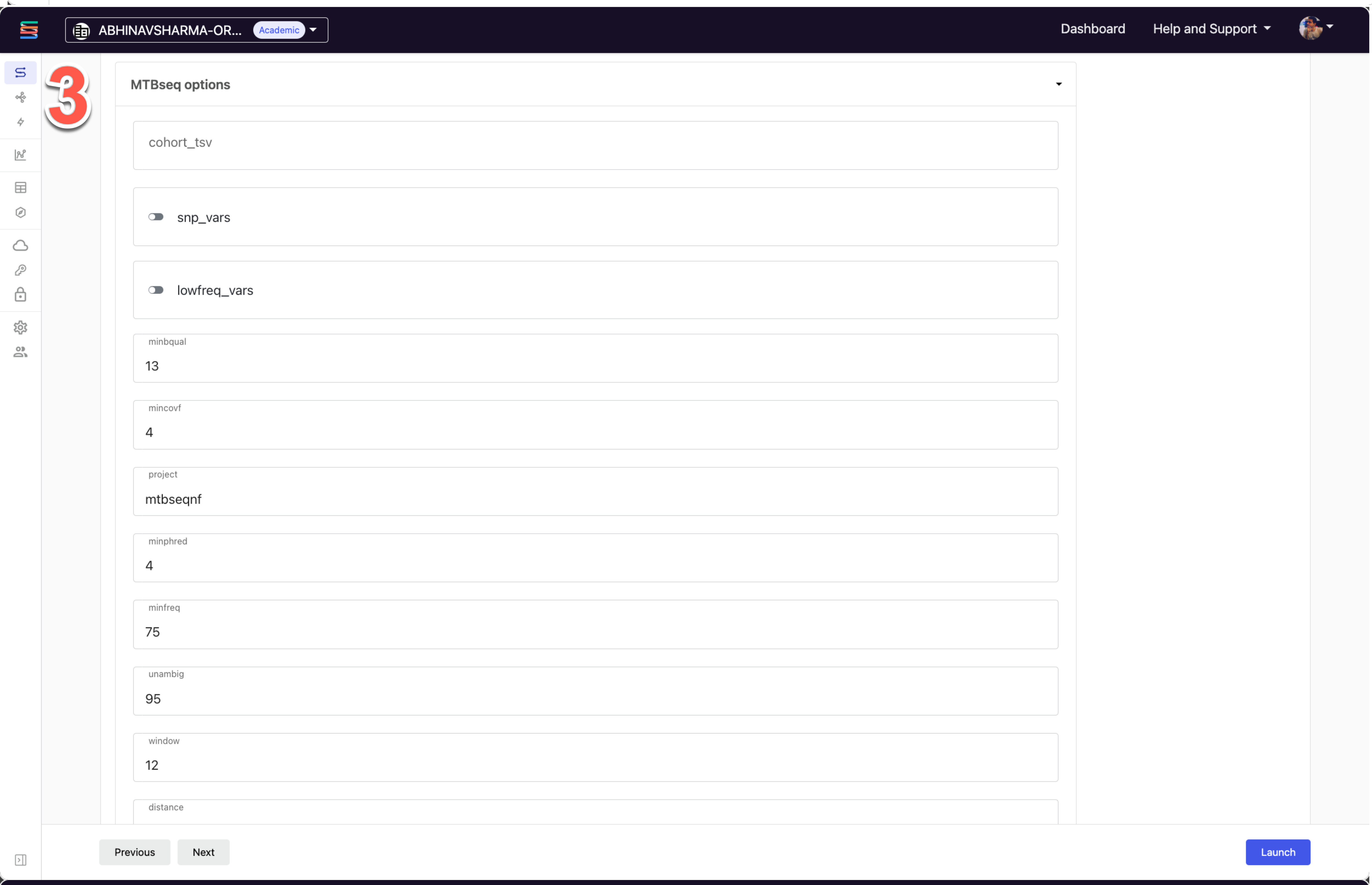
ABHINAVSHARMA-OR...
Academic
Dashboard
Help and Support
3
MTBseq options
cohort_tsv
snp_vars
lowfreq_vars
minbqual
13
mincovf
4
project
mtbseqnf
minphred
4
minfreq
75
unambig
95
window
12
distance
Previous
Next
Launch

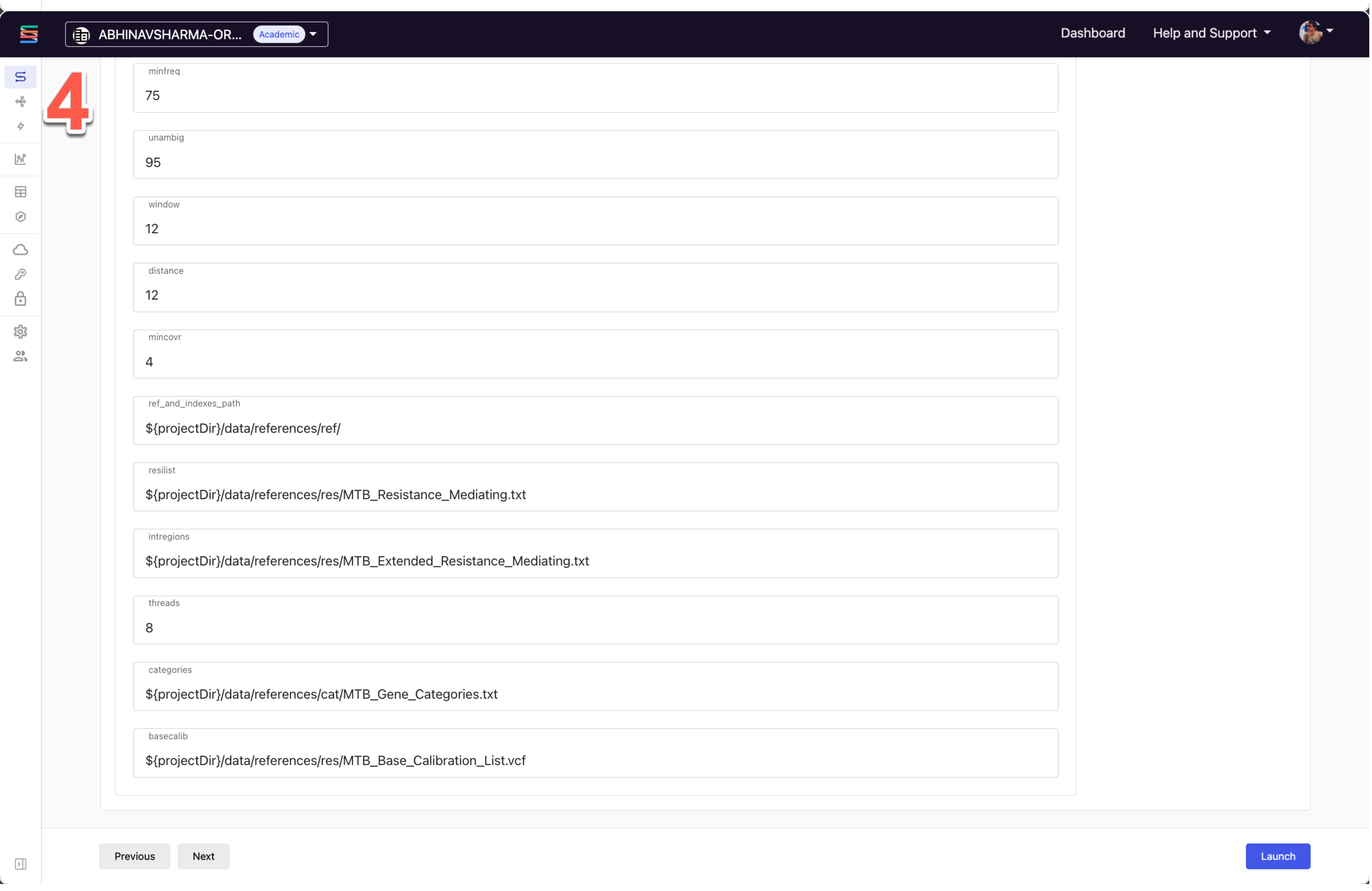
ABHINAVSHARMA-OR...
Academic
Dashboard
Help and Support
4
minfreq
75
unambig
95
window
12
distance
12
mincovr
4
ref_and_indexes_path
${projectDir}/data/references/ref/
resilist
${projectDir}/data/references/res/MTB_Resistance_Mediating.txt
intregions
${projectDir}/data/references/res/MTB_Extended_Resistance_Mediating.txt
threads
8
categories
${projectDir}/data/references/cat/MTB_Gene_Categories.txt
basecalib
${projectDir}/data/references/res/MTB_Base_Calibration_List.vcf
Previous
Next
Launch
